# Modified clinical decision rule for termination-of-resuscitation in cases of refractory out-of-hospital cardiac arrest

**DOI:** 10.1186/cc10882

**Published:** 2012-03-20

**Authors:** Y Goto, T Maeda, Y Goto, H Inaba

**Affiliations:** 1 Kanazawa University Hospital, Kanazawa, Japan; 2 Yawata Medical Center, Komatsu, Japan; 3 Kanazawa University Graduate School of Medicine, Kanazawa, Japan

## Introduction

Two international termination-of-resuscitation (TOR) rules for the emergency medical services (EMS) personnel have been proposed to identify nonsurvivors after out-of-hospital cardiac arrest (OHCA). The first is for use by responders providing basic life support (BLS) which includes three criteria: not witnessed by EMS personnel, no shocks are administered and no return of spontaneous circulation (ROSC). The other is for use by responders providing advanced life support (ALS) which adds two criteria: unwitnessed by a bystander and no bystander cardiopulmonary resuscitation. Simpler criteria as a universal TOR rule may be desirable for any level of EMS personnel. We performed this study to validate two TOR rules and a modified BLS TOR rule which includes three criteria: unwitnessed arrest, no shocks administered and no ROSC achieved before arrival at hospital for predicting refractory OHCAs.

## Methods

We analysed 289,769 OHCA adult patients with presumed cardiac causes, using a prospectively recorded nationwide Utstein-style database in Japan over 5 years (2005 to 2009). The primary endpoint was 1-month survival with unfavourable neurological outcome, or Glasgow-Pittsburgh cerebral performance category (CPC) scale = 3 to 5.

## Results

The overall rates of 1-month survival with CPC = 1 or 2 and collective 1-month survival were 2.55% and 5.22%, respectively. The incidences of misclassification in the BLS, ALS and modified BLS TOR rules for 1-month survival with CPC = 3 to 5 were 0.20%, 0.15% and 0.13%, respectively. The specificity (95% CI) in the BLS, ALS and modified BLS TOR rules for 1-month survival with CPC = 3 to 5 were 0.941 (0.935 to 0.946), 0.981 (0.978 to 0.984) and 0.972 (0.968 to 0.975), respectively. The area under the receiver operating characteristic curve in the BLS, ALS and modified BLS TOR rules for 1-month survival with CPC = 3 to 5 were 0.865, 0.654 and 0.765, respectively.

## Conclusion

We found that each TOR rule had high specificity (ability to predict survivors with favourable neurological outcome) and low misclassification rate as a universal TOR rule. The modified BLS TOR rule is simpler and as reliable as the other two rules. In Japan, as EMS providers are legally prohibited from terminating resuscitation in the field, the amendment of related laws and the establishment of national consensus would be necessary to apply these rules in the Japanese EMS system.
